# The Evolutionary History of Amino Acid Variations Mediating Increased Resistance of *S. aureus* Identifies Reversion Mutations in Metabolic Regulators

**DOI:** 10.1371/journal.pone.0056466

**Published:** 2013-02-12

**Authors:** Mia D. Champion, Vanessa Gray, Carl Eberhard, Sudhir Kumar

**Affiliations:** 1 Center for Evolutionary Medicine & Informatics, Biodesign Institute, Arizona State University, Arizona, United States of America; 2 Division of Pathogen Genomics, Translational Genomics Research Institute, Arizona, United States of America; Cairo University, Egypt

## Abstract

The evolution of resistance in *Staphylococcus aureus* occurs rapidly, and in response to all known antimicrobial treatments. Numerous studies of model species describe compensatory roles of mutations in mediating competitive fitness, and there is growing evidence that these mutation types also drive adaptation of *S. aureus* strains. However, few studies have tracked amino acid changes during the complete evolutionary trajectory of antibiotic adaptation or been able to predict their functional relevance. Here, we have assessed the efficacy of computational methods to predict biological resistance of a collection of clinically known Resistance Associated Mutations (RAMs). We have found that >90% of known RAMs are incorrectly predicted to be functionally neutral by at least one of the prediction methods used. By tracing the evolutionary histories of all of the false negative RAMs, we have discovered that a significant number are reversion mutations to ancestral alleles also carried in the MSSA476 methicillin-sensitive isolate. These genetic reversions are most prevalent in strains following daptomycin treatment and show a tendency to accumulate in biological pathway reactions that are distinct from those accumulating non-reversion mutations. Our studies therefore show that in addition to non-reversion mutations, reversion mutations arise in isolates exposed to new antibiotic treatments. It is possible that acquisition of reversion mutations in the genome may prevent substantial fitness costs during the progression of resistance. Our findings pose an interesting question to be addressed by further clinical studies regarding whether or not these reversion mutations lead to a renewed vulnerability of a vancomycin or daptomycin resistant strain to antibiotics administered at an earlier stage of infection.

## Introduction

Within a decade following the accidental discovery of penicillin G, the majority of known *S. aureus* isolates produced a ß-lactamase enzyme mediating resistance to the antibiotic [1]. This led to the development of a more effective antimicrobial (ß-lactamase-resistant ß-lactam methicillin), an effort that was counteracted by methicillin resistant *S. aureus* (MRSA) [1], [2]. The rapid and prolific emergence of resistance has diminished the effectiveness of every tested class of antibiotics to date, including vancomycin and daptomycin [1]–[3]. And the phenomenon of cross-resistance has emerged as an important mechanism in the development of pan-resistance to numerous classes of antimicrobials against *S. aureus* [4]. Reports of daptomycin-resistant *S. aureus* isolates evolving following exposure to vancomycin treatment are increasing in prevalence and are of great concern since these drugs are “last-line of defense” antibiotics against infection [5], [6].

Recent studies of several infectious pathogens have revealed how processes of horizontal gene transfer, recombination, and parallel evolution can lead to sequence variations that give rise to the progression of antibiotic resistance [7]–[10]. The sequential accumulation of multiple nonsynonymous mutations has also been described, and these acquired mutations can contribute directly to increasing virulence by promoting drug evasion or may act to lower the fitness costs of accumulating such variations (e.g. compensatory mutations) [7]–[9]. For example, specific genetic mutations in *S. aureus* that are associated with adaptation to vancomycin or daptomycin exposure have recently been reported, and these studies have also established that the progression of resistance in these isolates is accompanied by phenotypic alterations in virulence [6], [11], [12]. Lowered daptomycin susceptibility in a set of carefully selected isogenic clinical and laboratory *S. aureus* strains has been reported to be the consequence of specific mutations in the *mprF* gene encoding a membrane protein, the (*walk (yyG)*) gene encoding a sensor histidine kinase, and the *rpoB* gene encoding a RNA polymerase subunit [12]. Amino acid variants in several other proteins (*vraG, agrA, dltA, rpoB, yvqF*, and *stp1*) acting in multiple genetic pathways have been previously shown to reduce the susceptibility of *S. aureus* isolates to vancomycin treatment [6], [11], and are important regulators of cell wall synthesis and metabolic control [13]–[16].

Previously identified *S. aureus* mutations associated with increased resistance have not been evaluated in the context of evolution. In general, neutral evolutionary trends over time help to establish *a priori* expectations of functional effect because each position in each protein has undergone functional evaluation across evolution [17], [18]. As such, Evolutionary Permissible Alleles (EPAs) are predicted as functionally benign whereas damaging predictions are associated with significant adaptions such as those required for acquisition of resistance. Determining positional evolutionary rates of relative allele frequencies constitutes the basis of several *in silico* prediction methods that have been developed and commonly applied to studies of human genomes in order to provide a diagnosis of a given variation as functionally benign or damaging [19]–[21].

We have discovered that these prediction methods are significantly inaccurate when applied to diagnosing known RAMs. Furthermore, by tracing the evolutionary history of the false negative RAMs, we have found that these mutations are genetic reversions to conserved ancestral alleles that are carried in a phenotypically methicillin-sensitive isolate (MSSA476). Reversion RAMs are more prevalent in strains exposed to daptomycin treatment and show a tendency to accumulate in enzymes mediating certain reactions of complex biosynthetic pathways. Our findings should therefore be of interest to clinical researchers who have the means to evaluate the relevance of genetic reversions as potential drug targets during the course of treatment.

## Results

### Failure of Computational Methods to Accurately Predict Resistance Associated Mutations

Evaluation of whole genome *S. aureus* sequences includes efforts to identify non-neutral sequence changes that are adaptive in the presence of an antibiotic. We therefore adapted popular tools (PolyPhen, SIFT, and CAROL [19]–[21]) commonly used for this purpose and applied the *in silico* prediction methods to known RAMs.

**Figure 1:**
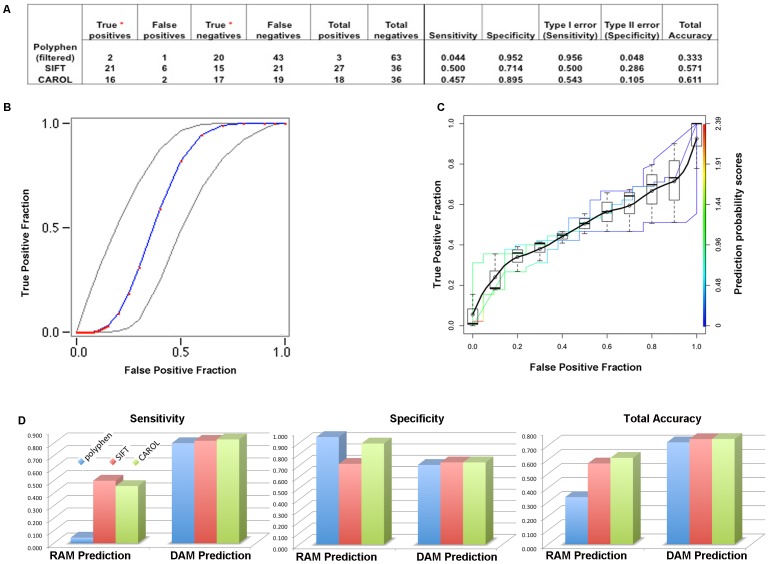
Estimation of the accuracy of predicting RAMs using three methods. The accuracy statistics of three methods in predicting StaphRAMs (A–C) is compared to the known accuracy of the same methods for predicting Human DAMs [21] (D) Maximum likelihood estimation of a binormal ROC curve with an asymmetric 95% confidence interval using 3 methods of prediction (B), with CAROL predictions representing the strongest evidence of a damaging prediction (C). The area of the ROC curve is 0.63 with a standard deviation of 0.06. Accuracy, sensitivity, and specificity were estimated to be 62.6%, 94.0% and 38.5% respectively. Red and blue represent the fitted ROC curve (B). Grey lines denote the 95% confidence interval of the fitted ROC curve (B). A boxplot of the spread estimate for all three methods is shown and ROCR curves for each method are colorized according to the prediction cutoffs (C).

Performance statistics for accurately predicting known *S. aureus* RAMs using three commonly used methods (PolyPhen, SIFT, and CAROL) [19]–[21] (Figure 1A–C) were compared to the accuracy statistics for predictions of Human Disease Associated Mutations (DAMs) [21] using the same computational methods (Figure 1D). Overall, the accuracy of the available tools in predicting RAMs is significantly lower in comparison to predictions of human DAMs (Figure 1D). In order to exclude the possibility that overrepresentation of certain subspecies in the sequence alignments was leading to allele frequency biases that were resulting in false negative predictions, the polyphen analysis was also run with a “filtered alignment” dataset that only included one representative sequence from each closely related bacterial species (Methods). This approach did not significantly improve the number of accurate predictions for the positive control dataset (Table 1). CAROL provides a weighted prediction dependent on PolyPhen and SIFT scores and consistent with the analysis of human variations, the overall prediction accuracy improves when all three tools are used in combination (Figure 1C), thus minimizing the effect of significant spread estimates observed when SIFT and PolyPhen are used independently (Figure 1C) [22], [23].

**Table 1:** Predictions of Functional Relevance For Positive Control RAMs [9], [10] Identified From Clinical or Laboratory-derived Isolates Following Exposure to Vancomycin or Daptomycin.

Vancomycin/Daptomycin Susceptible Strain Id of Each Pair/Series (P-MRSA) [9], [10]	Antibiotic Exposure of Clinical/Laboratory Isolate	Gene	Amino Acid position	MSSA (476) allele	MRSA (SAUSA300) allele	P-MRSA	Insensitive/Resistant Allele	Polyphen prediction (filtered alignments)	Polyphen score (filtered alignments)	Polyphen prediction (unfiltered alignments)	Polyphen score (unfiltered alignments)	SIFT prediction	SIFT score	CAROL prediction	CAROL score
A10102	Daptomycin	aroH/aroA	301	K	K	K	R	benign	0.334	benign	0.161	Tolerated	1.000	Neutral	0.160
		feoB	636	K	K	K	E	benign	Not Scored	benign	Not Scored	Deleterious	0.000	0	0.000
		mprF	295	S	S	S	L	benign	0.227	benign	0.414	Deleterious	0.040	Neutral	0.955
		*SA0754*	*139*	*G*	*G*	*S*	*G*	*benign*	*Not Scored*	*benign*	*Not Scored*	*Tolerated*	*1.000*	*0*	*0.000*
A9299	Daptomycin	era/RAS	185	H	H	H	Y	benign	1.432	benign	0.019	Deleterious	0.000	Deleterious	0.999
A5937	Vancomycin	lysP	290	L	L	L	F	benign	Not Scored	benign	Not Scored	Tolerated	0.370	0	0.000
		rpoB	481	H	H	H	Y	benign	0.046	possibly damaging	1.657	Deleterious	0.000	Deleterious	0.999
A5948	Laboratory-derived series, Daptomycin	cls2	33	T	T	T	N	benign	1.054	benign	0.122	Deleterious	0	Deleterious	0.999
		mnhC	110	A	A	A	E	benign	Not Scored	benign	Not Scored	Deleterious	0.000	0	0.000
		mprF	826	L	L	L	F	benign	Not Scored	benign	Not Scored	Deleterious	0.000	0	0.000
A6224	Vancomycin	dltA	38	S	S	S	R	benign	1.336	benign	1.037	Deleterious	0.040	Neutral	0.960
		tilS	128	M	M	M	I	benign	0.166	possibly damaging	1.883	Tolerated	0.170	Neutral	0.830
A6300	Vancomycin	hemL (duplicate copy)	48	Y	Y	Y	D	benign	0.508	benign	0.099	Deleterious	0.000	Deleterious	0.999
		rpsK	127	R	R	R	G	benign	Not Scored			Deleterious	0.010	0	0.000
		*drp35*	*83*	*S*	*S*	*N*	*S*	*benign*	*0.038*	*benign*	*0.892*	*Tolerated*	*0.850*	*Neutral*	*0.877*
		*hemL*	*48*	*D*	*D*	*G*	*D*	*benign*	*0.439*	*benign*	*0.235*	*Tolerated*	*1.000*	*Neutral*	*0.235*
		*purD*	*389*	*A*	*A*	*V*	*A*	*benign*	*Not Scored*	*benign*	*Not Scored*	*Tolerated*	*1.000*	*0*	*0.000*
A9635	Vancomycin	vraG	580	A	A	A	V	benign	0.166	benign	0.462	Deleterious	0.000	Deleterious	0.999
A8117	Daptomycin	asd	94	N	N	N	D	benign	0.608	benign	1.453	Deleterious	0.000	Deleterious	0.999
		pgsA	64	A	A	A	V	benign	0.957	benign	0.596	Deleterious	0.000	Deleterious	0.999
A8117	Laboratory-derived series, Vancomycin	walk	263	R	R	R	C	possibly damaging	1.629	benign	1.252	Deleterious	0.000	Deleterious	0.999
		*tcaR*	*69*	*S*	*S*	*I*	*S*	*benign*	*0.355*	*benign*	*0.532*	*Tolerated*	*1.000*	*Neutral*	*0.532*
A8796	Daptomycin	mprF	337	S	S	S	L	benign	0.585	benign	0.286	Deleterious	0.000	Deleterious	0.999
		*citZ*	*221*	*G*	*G*	*S*	*G*	*benign*	*1.032*	*benign*	*0.342*	*Tolerated*	*1.000*	*Neutral*	*0.342*
A8819	Daptomycin	cls2	60	F	F	F	S	benign	0.037	benign	0.849	Deleterious	0.000	Deleterious	0.999
		leuS	468	N	N	N	D	benign	1.348	benign	0.131	Deleterious	0.000	Deleterious	0.999
		mprF	345	T	T	T	I	benign	0.752	benign	0.501	Deleterious	0.010	Deleterious	0.989
		*mnaA*	*302*	*S*	*S*	*R*	*S*	*benign*	*1.043*	*benign*	*1.146*	*Tolerated*	*0.290*	*Neutral*	*0.710*
A9635	Vancomycin	rplC	7	G	G	G	V	benign	0.015	possibly damaging	1.538	Deleterious	0.000	Deleterious	0.999
A9719	Daptomycin	cls2	23	I	I	I	V	benign	1.375	benign	0.245	Tolerated	0.480	Neutral	0.421
		stp1	99	M	M	M	I	benign	0.995	benign	0.111	0	0.000	0	0.000
A9754	Daptomycin	*agrC*	*58*	*S*	*S*	*P*	*S*	*benign*	*1.23*	*possibly damaging*	*1.714*	*Tolerated*	*1.000*	*Neutral*	*0.000*
		vyqF	119	W	W	W	R	probably damaging	2.345	benign	0.416	Deleterious	0.000	Deleterious	0.999
		*bioF*	*175*	*A*	*V*	*I*	*V*	*benign*	*1.007*	*benign*	*0.452*	*Tolerated*	*1.000*	*Neutral*	*0.452*
		*purA*	*231*	*H*	*H*	*Y*	*H*	*benign*	*0.038*	*benign*	*0.892*	*Tolerated*	*1.000*	*Neutral*	*0.000*
		*rpoB*	*468*	*Q*	*Q*	*K*	*Q*	*benign*	*0.208*	*benign*	*0.066*	*Tolerated*	*1.000*	*Neutral*	*0.065*
		*walk*	*471*	*T*	*T*	*I*	*T*	*benign*	*0.439*	*benign*	*0.235*	*Tolerated*	*0.440*	*Neutral*	*0.468*
A9763	Daptomycin	cls2*	52	A	A	A	F	benign	0.022	probably damaging	2.391	Deleterious	0.000	Deleterious	0.999
		*SA1667*	*54*	*H*	*H*	*L*	*H*	*benign*	*0.911*	*benign*	*0.875*	*Tolerated*	*1.000*	*Neutral*	*0.875*
A9765	Daptomycin	*SA1291*	*239*	*V*	*V*	*A*	*V*	*benign*	*0.144*	*benign*	*1.355*	*Tolerated*	*0.700*	*Neutral*	*0.300*
A9781	Daptomycin	dprA/smf	166	V	V	V	A	benign	Not Scored	benign	Not Scored	0	0.000	0	0.000
		pdhA	169	A	A	A	T	benign	1.164	NA	NA	Not Scored	Not Scored	Not Scored	Not Scored
		rpoB	477	A	A	A	D	benign	0.035	benign	0.859	Deleterious	0.000	Deleterious	0.999
		*citZ*	*222*	*A*	*A*	*T*	*A*	*benign*	*0.212*	*benign*	*0.741*	*Tolerated*	*1.000*	*Neutral*	*0.741*
		*SA0248*	*342*	*G*	*G*	*C*	*G*	*benign*	*0.127*	*benign*	*0.983*	*Tolerated*	*0.450*	*Deleterious*	*0.982*

(*) indicates P-MRSA to VISA/DRSA amino acid changes according to publicly available sequences, and different from published AA changes. Identified reversion mutations are in bold, italic font.

**Figure 2:**
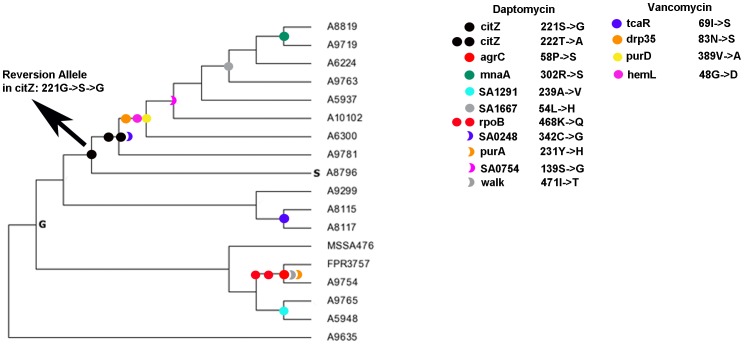
Reversion RAMs accumulate in strains exposed to daptomycin and in one copy of a multicopy protein. Ancestor states for each reversion RAM were determined and mapped onto subspecies phylogenetic reconstructions as described in the methods using MEGA5. Nodes of the tree where mutations occurred in Parental-MRSA (P-MRSA) isolates from patients prior to exposure to daptomycin or vancomycin treatments [11], [12] and reverted post-drug treatment to an allele also present in a Methicillin-Sensitive isolate (MSSA476) are annotated. A detailed evolutionary history for protein position 221 of the citZ protein in strain A8796 (treated with daptomycin) is provided as a model case. Evolutionary history analysis for each of the identified reversion alleles show that although reversion mutation accumulation biases seem to have occurred in certain strains, reversions to the allele present in a Methicillin-sensitive isolate (MSSA476) has occurred throughout the diversification of the lineage. In the case of the hemL protein, only one protein copy in strain A6300 has acquired the reversion mutation shown. A second hemL copy has acquired a Y→Y→D nonsynonymous mutation (not shown).

Given the low accuracy of prediction tools in identifying known RAMs, we traced the evolutionary history of the false negative cases and discovered that a significant number are reversion mutations to ancestral alleles, also carried in the genome of the MSSA476 methicillin-sensitive isolate (Figure 2).

### False Negative RAMs are Genetic Reversions to Alleles Carried in a Methicillin Sensitive isolate

Approximately 33% of the total positive control RAMs studied are genetic reversions to an allele carried in more ancestral *S. aureus* strains, including the MSSA476 isolate (Figure 2). Many reversions are also shared with another MRSA isolate lineage, USA300 (FPR3757) (Figure 2, Table 1)). A high percentage (>70%) of the RAM reversion mutations exhibit a significant association with non-neutral codons, and therefore are under more significant selective constraints mediating adaptation and fitness (Methods). Both polar and non-polar amino acids revert to MSSA alleles after drug treatment and almost all of them exhibit a neutral side chain charge (Table 1). Independent of the greater total number of variations analyzed from daptomycin-exposed strains, we find a significant presence of reversion allele mutations associated with daptomycin-exposed strains (Fisher's exact test, p-value≪0.01) in comparison to vancomycin-exposed strains (p-value>0.5) (Table I, Figure 2). Reversion mutations accumulate in protein sequences conserved in all strains examined and also arise in one copy of multi-copy proteins (e.g. HemL) (Figure 2). Notably, all of the identified revertant alleles fail to be accurately predicted as functionally damaging by the three methods (SIFT, PolyPhen, and CAROL), with the exception of the *agrC* and *SA0248* reversion RAMs that were accurately predicted by PolyPhen (*agrC*) or CAROL (*SA0248*) (Table 1). *AgrC* encodes a quorum sensing receptor previously shown to mediate *S. aureus* resistance to deformylase by accumulating mutations compensating for *fmt* mutations, which reduce bacterial growth rates [15], [24]. *SA0248* encodes a glycosyl transferase, which transfers sugar moieties to teichoic acids. Predictions from both SIFT and PolyPhen for the amino acid change present in *SA0248* following drug exposure were benign however, together their weighted scores gave a damaging prediction (Table 1). There were several cases of prediction conflict between PolyPhen and SIFT, which were more reliably resolved by implementation of CAROL rather than by selective filtering of sequence alignments (Methods, Table 1). Although in these cases, the instance of CAROL resolving to a deleterious prediction was less than 100% (Table 1). For humans, the frequency of nSNVs is directly correlated with evolutionary rate for a given position. The fastest-evolving positions are EPAs significantly more frequently than slower positions (81% vs. 53%, P≪0.01) [25]. Disease associated mutations in humans are significantly associated with slower evolving positions and only a small fraction (<10%) are EPAs [25]. We find that all of the *S. aureus* positive control RAMs occur at slower evolving nucleotide positions, however, 30–40% of these positions are within codons that are evolving at a faster rate (Methods) [26].

### Metabolic Regulators in Specific Biological Pathways Carry Reversion RAMs

**Figure 3:**
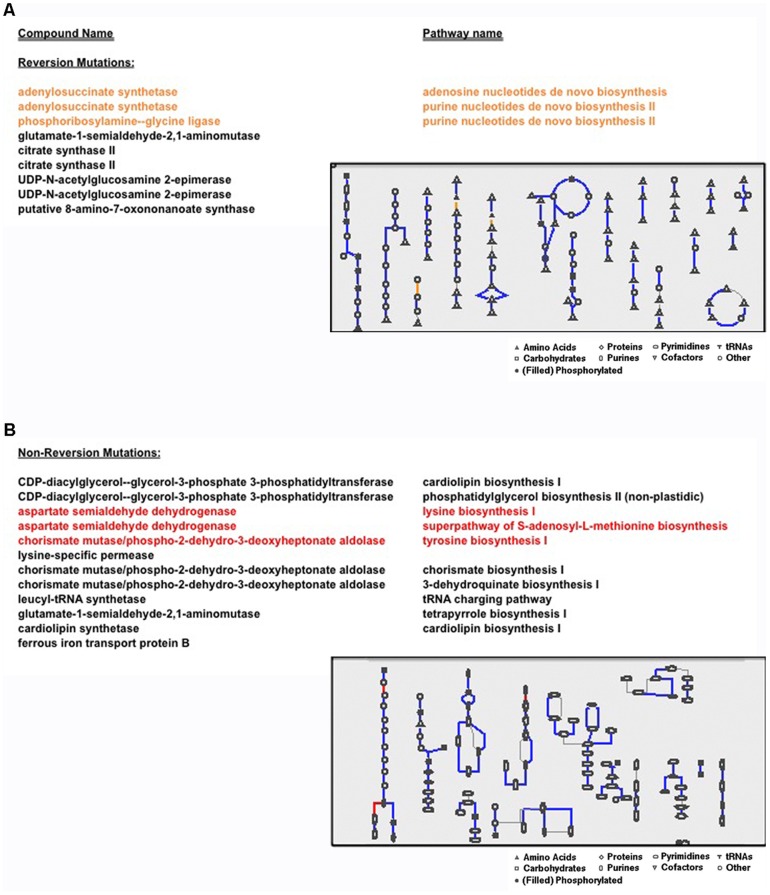
Prevalence of non-reversion and reversion mutations in complex biosynthetic metabolic pathways. Reversion (A) and non-reversion mutations (B) identified in protein sequences from all MRSA isolates exposed to drug treatment were mapped by homology to the *Staphylococcus aureus* metabolome [31] (mutations in compounds acting in Nucleoside/nucleotide Biosynthesis (A, orange) and Amino Acid Biosynthesis (B, red) are shown). Compounds accumulating mutations interact in complex pathway networks that involve highly conserved proteins (solid lines) as well as “pathway-holes” (light grey lines) where identified sequence or functional homologs have not yet been identified in the *Staphylococcus aureus* genomes. The propensity of different mutant types to accumulate in distinct biological pathways, and significant correlation of reversions arising in strains exposed to daptomycin, is suggestive of how exposure to different drugs likely presents evolutionary adaptation biases in metabolic regulation in order to minimize fitness cost while promoting the progression of antibiotic resistance.

A significant number of proteins carrying known RAMs are metabolic regulators. Interestingly, reversion mutations accumulate in enzymes mediating certain reactions of complex biosynthetic pathways whose intermediates are functionally involved with those that accumulate non-reversion mutations (Figure 3) [27]. The different reactions identified are involved in the following complex processes mediating the biosynthesis of: nucleosides/nucleotides, amino acids, aminoacyl-tRNA (“charged” tRNA), fatty acids and lipids, aromatic compounds, cofactors, prosthetic groups, and electron carriers (Figure 3). Specifically, reversion RAMs were identified in the adenylosuccinate synthetase and phosphoribosylamine-glycine ligase enzymes, both of which are involved in *de novo* biosynthesis of purines (Figure 3A).

Non-Reversion RAMs were not identified in enzymes primarily mediating nucleoside/nucleotide biosynthesis, but rather, accumulated in reactions central to amino acid synthesis (Figure 3B). It should be noted, however, that intrinsic to the nucleotide and amino acid biosynthetic metabolic processes are complex interconnected pathways that frequently share several key intermediates of reactions involving the transfer of nitrogen or one-carbon groups [28]. For example, purines and pyrimidines incorporate certain amino acids, or amino acid functional groups, and histidine synthesis requires part of a purine ring [28].

Enzymes with transferase activities required for amino group transfer (glutamate-1-semialdehyde-2,1-aminomutase) and amino acid metabolism (putative 8-amino-7-oxononanoate synthase) also carry reversion RAMs (Figure 3A). UDP-N-acetylglucosamine 2-epimerase and citrate synthase II enzymes both carry multiple reversion RAMs. In bacteria, UDP-N-acetylglucosamine 2-epimerase enzymes catalyze the reaction producing UDP-ManNAc, which is critical to the formation of the antiphagocytic capsular polysaccharide in pathogens such as *Streptococcus pneumoniae* [29]. Citrate synthases catalyze a condensation reaction involving acetyl coenzyme A, an important intermediate molecule of the Krebs Cycle and precursor of lipids and steroids [28]. Likewise, non-reversion RAMs were identified in enzymes involved in other reactions mediating fatty acid and lipid metabolism (Figure 3). Recent studies have shown that phospholipid synthesis in *S. aureus* accounts for their sensitivity to FASII inhibitors, which is unique in comparison to other pathogens such as *S. pneumoniae* [30].

## Discussion

In response to drug treatment, it is known that bacterial pathogens acquire multiple nonsynonymous mutations in proteins that either contribute directly to increasing virulence by promoting drug evasion or may act to lower the fitness costs of accumulating such variations (e.g. compensatory mutations) [7]–[9]. Our studies show that resistance associated mutations carried in either daptomycin or vactomycin insensitive isolates are in fact reversion mutations to alleles carried in a methicillin sensitive isolate. These reversion RAMs, therefore play a role (compensatory or otherwise) in mediating the continual progression of resistance to different antibiotic treatments. Our positive RAM control group does include reported compensatory mutations encoded by the *agrC, tcaR* and *walk* genes [6], [11]. Using our methods, two mutations in *walk* homologous protein sequences yielded different predictions regarding their functional relevance (Table 1). In a laboratory-derived strain exposed to vancomycin, a R263C mutation is predicted as damaging, whereas in a daptomycin-exposed strain the I471T change is benign. The variation that occurs at position 471 in the walk protein is a reversion to the MSSA476-allele. We also find that a K468Q RAM arising in the *rpoB* gene following daptomycin exposure is a reversion to the MSSA476-allele and is predicted as benign, in comparison to two RAMs in *rpoB* homologous translations that are accurately predicted as damaging in strains following either exposure to daptomycin or vancomycin (Table 1). Point mutations in the β subunit of RNA polymerase encoded by *rpoB* are known to render bacterial pathogens, such as *S. aureus* and *Mycobacterium tuberculosis*, resistant to rifampin treatment [31], [32]. Similar to studies of *E. coli*, a recent study of rifampin-resistant clinical *M. tuberculosis* isolates has shown that compensatory evolution in other RNA polymerase subunits encoded by *rpoA* and *rpoC*, reduces fitness cost in comparison to their susceptible counterparts [9], [33]–[35]. In both *E. coli* and *M. tuberculosis*, mitigation of the deleterious effects of a RAM by accumulation of compensatory mutations at independent sites has been established, however, it is not known whether these compensatory mutations are genetic reversions to more ancestral alleles present in the population [9], [33].

Similar to *agrC*, it is possible that other reversion RAMs identified in our studies are also compensatory in function, or general suppressors that may reduce fitness costs, even though their role has mainly been previously described relative to the progression of resistance [11], [12]. A previous study of clinical menadione-auxotrophic small-colony variant isolates of *S. aureus* that exhibit reduced gentamicin susceptibility, identified several sequence variations in the *menB* gene [36]. In one studied strain, growth-compensated mutants carrying genetic reversions and intragenic second-site mutations arose in the SCV population [36]. Compensatory roles of reversion mutations may also extend beyond one gene, or protein complex, and include functional pathways. For example, additional candidate compensatory mutations in S. aureus may include mutations in proteins with closely related dependencies in the same biological pathway (pgsA and cls2, Table 1). Or, may depend on more complicated interactions such as those we observe with the accumulation of reversion mutations in enzymes mediating certain reactions of complex biosynthetic pathways whose intermediates are functionally involved with those that accumulate non-reversion mutations (Figure 3). In addition, the significant correlation of reversions arising in strains exposed to daptomycin is suggestive of how exposure to a new antibiotic treatment may introduce reversion/non-reversion mutation acquisition biases in biological pathways important for fitness maintenance while promoting the progression of antibiotic resistance. It is unknown whether or not the reversion mutations identified by our studies also confer phenotypic sensitivity to antibiotics administered at an earlier stage of infection. In conclusion, future studies of RAMs in the context of evolution might therefore be applied to prioritize genetic variations for further clinical studies by profiling those that likely contribute to the progression of resistance by mediating adaptation to drug-exposure from reversion mutations acting to maintain the overall fitness of the organism. This type of approach would allow for the analysis of all predicted functional effects of genome-wide variations over the course of antibiotic treatments and therefore, would be advantageous in comparison to current prediction methods. Our findings that adaptation to incremental drug exposures includes the accumulation of methicillin-sensitive reversion mutations suggest the potential effectiveness of select multi-drug cocktails that target both resistance and fitness during the course of *S. aureus* adaptation.

## Methods

### Positive and negative control groups of amino acid variations

A total of 47 amino acid variations previously identified as conferring lowered susceptibility or resistance of MRSA isolates following exposure to either vancomycin or daptomycin were used as positive controls for testing computational prediction algorithms [11], [12] (Table 1). A total of 21 nonsynonymous Single Nucleotide Variations (nSNVs) between Parental-MRSA (P-MRSA) isolates from patients prior to exposure to daptomycin or vancomycin treatments [11], [12] and FPR3757 (MRSA) isolates at homologous positions not indicated by previous studies to mediate resistance were used as the negative control group for testing the efficacy of the prediction algorithms for bacterial mutations (Figure 1A–C).

### Predicting the functional effects of amino acid variations

Amino acid variations identified in resistant clinical and laboratory isolates and the susceptible strain pair allele (reference sequence) were used to assay for damaging predictions (Table I). Additional and independent analysis runs were done using either the FPR3757 USA300 MRSA whole genome sequence corrected for sequencing errors [37], or the publicly available MSSA476 genome as a reference [38] (data not shown). Closely related homologous protein sequences were retrieved from the NCBI database (unfiltered) and aligned using MUSCLE [39]. Comparison PolyPhen predictions were also done using alignments of one representative sequence from each publicly available species of *Staphylococcus, Listeria, Macrococcus*, and *Bacillus* (filtered). Custom python scripts were used to run a prediction pipeline including PolyPhen, SIFT, and CAROL and predictions were calculated as described in [19]–[21]. Performance statistics for the three methods was done as described in [21] (Figure 1A), and using JROCFIT and JLABROC4 (Figure 1B), as well as customized R scripts for the ROCR package (Figure 1C) for comparative calculations of Receiver Operating Characteristic (ROC) curves [21], [23].

### Evolutionary Rates and Phylogenetic Analysis

Sequence alignments were used to assess overlap of homologous RAM positions and codons with 2- or 4-fold degenerate sites in MEGA5 [26]. Ancestor states for each reversion RAM were determined and mapped onto subspecies phylogenetic reconstructions using MEGA5 [26]. Genome alignments of homologous coding sequences were used to identify four-fold degenerate sites. These sites were used to build a tree using the Maximum Likelihood method based on the data specific model using MEGA5 [26], [40]. The bootstrap consensus tree inferred from 1000 replicates is taken to represent the evolutionary history of the taxa analyzed. Unimetric branch lengths are shown to optimize visualization of ancestral relationships. Initial tree(s) for the heuristic search were obtained as described in the MEGA5 program [26]. A discrete Gamma distribution was used to model evolutionary rate differences among sites (5 categories (+*G*, parameter = 0.1000). The rate variation model allowed for some sites to be evolutionarily invariable ([+*I*], 73.6551% sites). The tree is drawn to scale, with branch lengths measured in the number of substitutions per site. The analysis involved 18 nucleotide sequences. All positions containing gaps and missing data were eliminated. There were a total of 50440 positions in the final dataset. Evolutionary analyses were conducted in MEGA5 [26]. Homologous sequence alignments for each individual protein were mapped onto the subspecies tree to trace the ancestral states for the reversion RAMs.
